# Digital psychiatry in major depression and suicidality: emotion tracking, smart monitoring, and AI-supported care

**DOI:** 10.1192/j.eurpsy.2026.10293

**Published:** 2026-07-31

**Authors:** J. Lopez-Castroman

**Affiliations:** Psychiatry, University of Santiago de Compostela, Santiago de Compostela, Spain

## Abstract

**Abstract:**

Major depressive disorder (MDD) and suicidal behavior are characterized by rapid emotional and behavioral changes that are often missed by standard, episodic clinical assessments. Digital psychiatry offers tools to observe these dynamics over time, supporting earlier detection of clinical worsening and more timely intervention.

This presentation draws on clinical research using ecological momentary assessment (EMA) and passive smartphone monitoring in patients with depression and suicidal behavior. We show how real-time emotion tracking—capturing affective intensity, variability, and reactivity—provides clinically meaningful information that complements routine symptom scales and suicide risk evaluations. Passive behavioral indicators allow ongoing monitoring of vulnerability with minimal patient burden.

We also discuss approaches for integrating longitudinal emotional and behavioral data to identify individual patterns associated with relapse and escalation of suicidal risk, with an emphasis on clinical interpretability and usability. We will address practical considerations for implementation in routine care and future directions toward more personalized, continuous, and proactive management of MDD and suicidality.

**Disclosure of Interest:**

None Declared

